# Assessing the role of mentors in mitigating burnout and enhancing professional development in medical education

**DOI:** 10.5116/ijme.659b.d08c

**Published:** 2024-01-25

**Authors:** Kingthong Anurat, Sorawut Thamyongkit, Samart Pakakasama, Sutida Sumrithe

**Affiliations:** 1 Chakri Naruebodindra Medical Institute, Faculty of Medicine Ramathibodi Hospital, Mahidol University, Thailand; 2 Department of Pediatrics, Faculty of Medicine Ramathibodi Hospital, Mahidol University, Thailand

**Keywords:** Burnout, medical student, mentorship, mentoring behaviours, professional identity

## Abstract

**Objectives:**

To assess the correlation between mentor behaviours and medical student burnout and their professional development within medical education.

**Methods:**

A cross-sectional study using convenience sampling was conducted among third-, fifth-, and sixth-year medical students (N=307). Participants voluntarily completed anonymous online questionnaires measuring the Mentor Behavior Scale, the Maslach Burnout Inventory-Student Survey, and the Professional Self-Identity Questionnaire. Multivariate regression analysis was performed to analyse relationships between student burnout, mentor behaviours and their impact on professional development.

**Results:**

Among participants, 26% (N=80) experienced burnout, which was significantly associated with lower competency support (OR = 2.0, 95% CI: 1.1-3.5, p = 0.016), medication use (OR = 2.1, 95% CI: 1.1-4.0, p = 0.029), and a lower Grade Point Average (OR = 3.3, 95% CI: 1.6-6.9, p = 0.001) compared to non-burnout students. In the development of professional identity, a high level of mentor relationship structure had statistically significant associations with higher scores in key domains of the Professional Self-Identity Questionnaire, including teamwork (OR = 3.9, 95% CI: 1.5-9.9, p < 0.01), communication (OR = 3.4, 95% CI: 1.5-7.7, p < 0.01), ethical awareness (OR = 3.3, 95% CI: 1.4-8.0, p < 0.01), and record use (OR = 2.8, 95% CI: 1.2-6.5, p < 0.05).

**Conclusions:**

The impact of mentor behaviours on medical students is evident. Enhancing mentorship by addressing specific mentor behaviours can improve programme quality. Future research should explore the long-term effects and strategies for effectively implementing targeted enhancements in mentor behaviours.

## Introduction

Mentoring is an essential process in which an experienced mentor guides and supports a mentee in achieving personal goals, enhancing competence, and nurturing professional identity.^1^^-^^3^ In medical education, mentoring is vital for students to meet professional standards, offering constructive feedback and serving as positive role models. This practice yields advantages not only for the mentees but also for the mentors themselves, fostering their personal and professional development and augmenting their capacities in leadership and teaching capabilities. Furthermore, effective mentoring has been shown to improve student retention and recruitment, thereby conferring benefits upon medical institutions.^4^

The medical learning environment presents numerous stressors, adversely affecting medical students.^5^ Previous studies indicated that 27-75% of medical students have exhibited symptoms of burnout.^6^^-^^10^ The primary factors linked to burnout were identified as the learning and work environments.^6^^,^^7^^,^^11^ The implementation of a mentorship programme demonstrated numerous benefits such as stress, anxiety, and depression reduction.^12^ Specifically, in the previous study regarding burnout, the medical students who participated in the mentorship programme reported a significant increase in personal accomplishment scores on burnout questionnaire.^13^ Mentorship programmes also play a crucial role in fostering the professional growth of medical students. Through role modelling and constructive feedback, mentors could help mentees cultivate their professional identity. It is the initial step to help students prepare themselves for professional roles.^14^

The success of a mentorship programme relies on various factors, among which the quality of the mentor-mentee relationship plays a major influence. This relationship is determined by multiple factors, including mentor's supportive behaviours.^2^ However, limited research has explored how mentors' supportive behaviours specifically influence medical students.

Our study aims to investigate the association between volunteer mentors' behaviours, as perceived by medical students, and their potential influence on medical students' experiences, including burnout and the development of their professional identity.

## Methods

### Context

The Faculty of Medicine Ramathibodi hospital has provided a medical degree programme for 58 years. The curriculum consists of pre-clinical (Year 1-3) and clinical (Year 4-6) phases. Currently, this programme admits 212 new students annually and provides longitudinal mentoring for every student. Volunteer mentors were randomly assigned to groups of five students, starting in Year 2 and continuing their mentorship with the same mentees until Year 6. All mentors receive training in essential mentoring skills, including effective communication, active listening, feedback, and goal-setting. While fulfilling their roles as mentors, participants in the programme engaged in regular meetings designed to provide support and instruction prior to each activity, as well as to facilitate the assessment and reflection upon their previous mentoring activities. In the pre-clinical years, initiatives promoting Professional Identity Formation (PIF) encompass early clinical exposure, which includes visits to patient wards and operating rooms, practicing communication skills during interactions with actual patients, and conducting physical examinations under the supervision of mentors. Psychological support and personal growth activities take place within mentor-led retreat sessions, fostering meaningful mentor-mentee interactions. These retreats cultivate an environment in which mentors engage in active listening, offer constructive feedback, and facilitate goal-setting within a supportive atmosphere. After the end of each academic year, this mentor program evaluation was conducted to assess the quality of mentorship program.

### Study design and participants

This cross-sectional study was conducted during the 2021 academic year at Faculty of Medicine Ramathibodi Hospital, Mahidol University. Participants completed online questionnaires using convenience sampling. Sample size for estimating a finite population proportion from n4Studies Plus resulted in a minimum requirement of 205 participants out of a total of 593 students.^15^ An additional 20% was included in the sample size to accommodate potential missing data, thus a total sample size was 257. Third-year medical students responded to two questionnaires: the Mentor Behavior Scale (MBS) and Maslach Burnout Inventory-Student Survey (MBI-SS). Fifth- and sixth-year medical students, who had experienced real workplace environments, completed these two questionnaires along with the Professional Self-Identity Questionnaire (PSIQ). The inclusion of the PSIQ in the fifth- and sixth-year aimed to assess their evolving professional self-identity due to their exposure to real workplace environments. Participants with incomplete responses to required questions were excluded from the study.

In this study, informed consent was obtained from all third-, fifth-, and sixth-year medical students prior to their participation. We employed convenience sampling, representing 51.8% (n=307) of the total students in the three classes (n=593). A total of 307 participants voluntarily completed the questionnaires and were included in the analysis. Among the participants, 47.9% (n=147) were male, and 52.1% (n=160) were female. Specifically, the sample comprised 165 third-year medical students, 43 participants from the fifth-year, and 99 from the sixth-year.

Our study was approved by Ethics Committee of the Institutional Research Committee of Ramathibodi (COA. MURA2021/558). All participants volunteered willingly, and strict measures were in place to ensure the anonymity of all participants throughout the research process. Consequently, the researchers were unable to identify respondents, minimizing the potential for selection bias.

Baseline characteristics, including gender, underlying disease, medication usage, Grade Point Average (GPA), duration of sleep, duration of exercise, extracurricular activities, and club activities, were collected. GPA was categorized into two groups for ease of interpretation: the low GPA group (GPA < 3.00) and the high GPA group (GPA ≥ 3.00). Detailed baseline characteristics of the participants are presented in Table 1.

### Data collection

Data for this study was collected through online questionnaires administered from January to April 2022. Participants were selected using convenience sampling, and prior to completing the questionnaires, all participants were provided with detailed information about the research objectives and assured of their voluntary participation rights. In order to mitigate non-response bias, various methods were employed to encourage participation in this study, including sending information via emails, promoting engagement in well-attended classes and post-examination sessions.

**Table 1:** Baseline characteristics (N=307)

Baseline characteristics		N (%)
Gender		
	Male	147 (47.9)
	Female	160 (52.1)
Underlying diseases (N=304)		
	Underlying diseases (Allergic rhinitis, PCOS, asthma, G6PD, ADHD, Crohn’s disease, gout, nasopharyngeal cancer, migraine, MDD, DM, narcolepsy)	77 (25.3)
	No underlying disease	227 (74.7)
Medication usage		
	Medication usage (Antihistamine, allopurinol, methylphenidate, OCP, ICS, Sertraline, fluoxetine, abilify, armodafinil, metformin)	53 (17.3)
	No medication usage	254 (82.7)
GPA (N=306)		
	2.00 - 2.49	6 (2)
	2.50 - 2.99	31 (10.1)
	3.00 - 3.49	137 (44.8)
	3.50 - 4.00	132 (43.1)
Duration of sleep per night (hours)		
	0 - <4	2 (0.7)
	4 - <6	106 (34.5)
	6 - <8	173 (56.4)
	≥ 8	26 (8.5)
Duration of exercise per week (minutes) (N=306)		
	0 < 50	128 (41.8)
	50 - <100	73 (23.9)
	100 - <150	47 (15.4)
	≥ 150	58 (19)
Extracurricular activities		
	Extracurricular activities	252 (82.1)
	No extracurricular activities	55 (17.9)
Club activities		
	Club activities	169 (55)
	No club activity	138 (45)

PCOS: Polycystic ovary syndrome; G6PD: glucose-6-phosphate dehydrogenase deficiency; ADHD: Attention deficit hyperactivity disorder, MDD: Major depressive disorder; DM: Diabetes mellitus; OCP: Oral contraceptive pill; ICS: Inhaled corticosteroids; GPA: Grade point average

### Research instruments

All questionnaires used in this study were authorized for use by their respective developers.

### Mentor Behavior Scales (MBS)

In this study, we employed the Mentor Behavior Scale (MBS) questionnaire, which comprises 15 Likert-scale items assessing four domains: Mentor Relationship Structure, Engagement, Autonomy Support, and Competence Support. The MBS exhibited robust psychometric properties, having undergone validation and reliability testing, with an RMSEA of 0.066 at Time 1 and 0.079 at Time 2, and an ordinal coefficient alpha exceeding 0.7 in all domains, except the autonomy support domain.^16^^-^^17^ The English version of this questionnaire was suitable for our research. We adopted the mentor behaviour profile category from a previous study.^2^ After reversing autonomy support scores, the optimal mentor behaviour profile consisted of high scores in all domains, and the controlling mentor behaviour profile had high scores in three domains, except for low scores in the autonomy support domain.

### Maslach Burnout Inventory-Student Survey (MBI-SS)

The Maslach Burnout Inventory survey, consisting of 15 Likert scale items, is a widely recognized tool used to assess burnout across various professions, including physicians, nurses, and medical students.  A Thai version of this questionnaire has already been translated and demonstrated acceptable interrater reliability, with a Kappa coefficient of 0.83. Confirmatory factor analysis further supported its validity, showing good fit indices.^18^ The identification of the burnout group was based on elevated scores in emotional exhaustion and depersonalization, alongside reduced scores in personal accomplishment, providing a comprehensive framework for assessing burnout in the studied population.

### Professional Self-Identity Questionnaire (PSIQ)

Professional self-identity questionnaire (PSIQ), English version, was employed to determine the professional development in fifth-and sixth-year medical students. This questionnaire comprises nine Likert scale items and has undergone content validity and reliability testing, yielding a Cronbach's alpha of 0.93.^14^ These nine items of PSIQ reflected professional self-identity formation in 9 domains. It included teamwork (PSIQ1), communication (PSIQ2), conducting assessment (PSIQ3), cultural awareness (PSIQ4), ethical awareness (PSIQ5), using records (PSIQ6), dealing with emergencies (PSIQ7), reflection (PSIQ8), and teaching (PSIQ9). Each item is scored on a scale ranging from 1 to 6. Participants were classified into two groups based on their scores: the low professional group (mean score ≤4) and the high professional group (mean score >4).

### Statistical analysis

The data analysis was performed using Stata Version 17. Categorical data were reported in terms of frequency and percentage, while continuous data were presented as either mean and standard deviation or median and range, depending on the data distribution characteristics. The comparison of baseline characteristics and study factors between groups of interest (burnout versus non-burnout groups and low versus high professional groups) was carried out using the Chi-square or Fisher’s exact test for categorical variables and the T-test or Mann-Whitney U test for continuous variables. Variables with p-values less than 0.1 in the univariate analysis were considered for inclusion in subsequent steps. The forward selection method was employed to select the appropriate model, and multiple logistic regression analysis was used to determine the associations between mentor behaviour domains and both burnout and each domain of professional self-identity formation in medical students. Odds ratios with 95% confidence intervals (CI) were estimated in the multiple logistic regression analysis. All statistical analyses were two-tailed, and a p-value of less than 0.05 was considered statistically significant.

## Results

### Mentor Behavior Scale scores

The scores were classified into three categories: high, moderate, and low, with the cut-off points for these categories referenced from a prior study.^19^ According to medical students' perspectives, the overall mean scores (sum of scores) were high in three out of four domains: mentor relationship structure, engagement, and competency support (Table 2).

**Table 2:** Mentor behavior scale (MBS) scores from medical students’ perspectives (N=307)

Domain	Min	Max	Mean sum score (SD)	Interpretation
Mentor relationship structure (Summation score of MBS 1-8) (high>31, moderate 24-31, low <24)	8	40	33.8 (6.0)	High score
Engagement (Summation score of MBS 9-10) (high>7, moderate 6-7, low <6)	2	10	8.5 (1.6)	High score
Autonomy support (Summation score of MBS 11-12) (high>7, moderate 6-7, low <6)	2	10	5.2 (2.1)	Low score
Competency support (Summation score of MBS 13-15) (high>11, moderate 9-11, low <9)	3	15	12.4 (2.4)	High score

### Burnout status in medical students

Out of the total 307 participants, 80 individuals (26%) were identified as experiencing burnout according to the Maslach Burnout Inventory-Student Survey. Multivariate analysis revealed several factors significantly associated with medical students' burnout. Participants reporting medication usage were found to be associated with burnout (OR = 2.1, 95%CI: 1.1-4.0, p = 0.029). Medical students with a low GPA (GPA<3.00) were also significantly associated with burnout (OR = 3.3, 95%CI: 1.6-6.9, p = 0.001). Additionally, low to moderate levels of competency support from mentors were significantly associated with burnout in medical students (OR = 2.0, 95%CI: 1.1-3.5, p = 0.016).

Given potential variations in curricula and experiences among medical students in different years, a subgroup analysis was conducted, focusing on participants from the pre-clinical year (third-year medical students) and those in the clinical years (fifth- to sixth-year medical students). Factors associated with burnout in pre-clinical year medical students included a low to moderate level of competency support from mentors (OR = 2.4, 95% CI: 1.1-5.0, p = 0.023) and a low GPA (OR = 10.0, 95% CI: 3.3-30.2, p < 0.001). However, in clinical years medical students, only the low to moderate level of mentor relationship structure was significantly associated with burnout (OR = 3, 95% CI: 1.3 to 7.2, p = 0.014) (Table 3).

**Table 3:** Multivariate subgroup analysis of factors associated burnout in pre-clinical medical student (n=165) and clinical medical students (n=142)

Factor		OR (95%CI)	p-value
Pre-clinical year			
Competency level			
	Low-moderate (score <=11)	2.4 (1.1-5.0)	0.023*
	High (score>11)	1	
GPA			
	2.00 - 2.99	10 (3.3-30.2)	0.000*
	3.00 - 4.00	1	
Clinical year			
Mentor relationship structure level			
	Low-moderate (score <=31)	3 (1.3-7.2)	0.014*
	High (score>31)	1	

*Significant at p < 0.05

### The professional self-identity formation in medical students

A total of 142 participants, consisting of fifth- and sixth-year medical students, completed the nine-item Professional Self-Identity Questionnaire (PSIQ). In the multivariate analysis, a high level of mentor relationship structure was significantly associated with high professional scores in the teamwork, communication, ethical awareness, and the use of records domains (Table 4). Additionally, a high level of autonomy support was significantly associated with a high professional score in the communication domain (OR = 4.3, 95% CI: 1.1-16.0, p = 0.031). Furthermore, a high level of competency support was significantly associated with a high professional score in the conducting assessment domain (OR = 5.8, 95% CI: 2.1-16.4, p = 0.001). However, no mentor behaviours were found to be significantly associated with the high professional group in the domains of cultural awareness, dealing with emergencies, reflection, and teaching (p > 0.05).

## Discussion

While mentorship programs have demonstrated benefits across various professions, limited research has explored the specific characteristics of mentors that alleviating burnout and cultivating professional identity among medical students. In this study, we utilized the Mentor Behavior Scale (MBS) questionnaire, which is grounded in the sociomotivational theoretical model^16^ to evaluate mentor behaviours within our mentorship programme and identify those most beneficial for medical students in achieving their professional goals. Our findings indicated high scores in mentor relationship structure, engagement, and competency support, while autonomy support received lower scores (Table 2). Notably, mentors tended to exhibit controlling profiles, consistent with a prior Malaysian study,^19^ potentially influenced by cultural similarities. These insights offer guidance for program improvement, underscoring the need for increased autonomy support for medical students.

**Table 4:** Mentor behavior scale and professional self-identity formation in medical students (after adjusted with significant baseline characteristics) (N=142)

Domain of PSIQ	Domain of MBS	OR (95% CI)	p-value
PSIQ 1. Teamwork	Mentor relationship structure (high score)	3.9 (1.5-9.9)	0.004*
PSIQ 2. Communication	Mentor relationship structure (high score)	3.4 (1.5-7.7)	0.004*
	Autonomy support (high score)	4.3 (1.1-16.0)	0.031*
PSIQ 3. Conducting assessment	Competency support (high score)	5.8 (2.1-16.4)	0.001*
PSIQ 5. Ethical awareness	Mentor relationship structure (high score)	3.3 (1.4-8.0)	0.008*
PSIQ 6. Using records	Mentor relationship structure (high score)	2.8 (1.2-6.5)	0.016*

*Significant at p < 0.05

Burnout is a recurring issue for medical students, often arising from challenging learning environments, heavy workloads, and examination-related stress.^6^^,^^7^^,^^11^^,^^20^ In our study, 26% of medical students experienced burnout, a rate in line with previous research findings ranging from approximately 27% to 75%.^6^^-^^10^ We identified a low GPA was associated with burnout, aligning with previous research in Saudi Arabia.^21^ Additionally, we observed that a low to moderate level of competency support from mentor was significantly linked to burnout. This implies that a high level of competency support might be a valuable predictor for reducing burnout. Subgroup analysis revealed that competency support domain remained particularly important for third-year medical students, suggesting that they benefit from positive reinforcement, regardless of their success or failure experiences. In contrast, clinical year medical students seemed to require a robust mentor relationship structure that allows them to share concerns and receive constructive feedback in order to prevent burnout.

Professional self-identity is crucial for the success of medical students in their professional development. Our study revealed how mentor behaviours not only mitigate burnout but also nurture a professional self-identity in medical students. Specifically, a strong mentor relationship structure was associated with various dimensions of professional self-identity, such as teamwork, communication, ethical awareness, and the use of records. Furthermore, high levels of competency support and autonomy support from mentors were linked to proficiency in assessments and effective communication, respectively.

Previous research has underscored the importance of factors such as the availability of mentors, the presence of role models, and the provision of constructive feedback in influencing professional self-identity formation.^22^^-^^25^The findings of this study affirm that mentoring programs significantly contribute to the cultivation of professional self-identity. Moreover, our study enriches the body of knowledge by pinpointing specific mentor behaviours that particularly facilitate the development of a strong professional self-identity among medical students.

### Limitations and future research

This study makes a substantial contribution to the exploration of the relationship between mentor behaviours, as assessed through the MBS questionnaire, and their impact on the mental well-being and professional self-identity formation of medical students. The findings offer valuable insights into specific mentor behaviours that can benefit medical students and provide guidance for mentors looking to enhance their mentoring skills. However, it is essential to acknowledge and address several limitations in this study. Firstly, the absence of a standardized evaluation of mentors' capabilities in guiding and supporting medical students may limit the generalizability of the study's findings to other mentorship programs. However, it is worth noting that our mentorship program did include training sessions, feedback from student satisfaction, and senior consultants for all participating faculty members. Secondly, the use of self-administered questionnaires may introduce recall bias and social desirability bias, and the assessment of mentor behaviours relied solely on the perspectives of medical students. Thirdly, important external factors, such as curriculum design, the learning environment, and workplace-based learning, were not considered in this study. These factors could potentially influence the formation of professional self-identity of medical students. Lastly, this study employed a cross-sectional design, which implies that the results only establish associations between mentor behaviours, medical student burnout, and professional identity formation. Causality cannot be determined based on this single-point data collection. Future research should consider conducting a longitudinal prospective cohort study that incorporates standardized evaluations of mentor abilities to further investigate these relationships.

## Conclusions

This study has demonstrated that specific mentor behaviours, such as competency support and mentor relationship structure, are associated with a reduced risk of burnout among medical students. Furthermore, mentor behaviours including mentor relationship structure, autonomy support, and competency support from mentors play a positive role in promoting the professional self-identity formation of medical students. These findings offer valuable insights for enhancing the effectiveness of mentorship programmes and provide valuable guidance for mentors seeking to enhance their mentoring skills.

### Acknowledgments

The authors would like to express sincere gratitude to the Faculty of Medicine at Ramathibodi Hospital, Mahidol University, for generously providing the opportunity and funding to undertake this research project. Special appreciation is extended to Associate Professor Sasivimol Rattanasiri and Mr Chanatpon Aonnuam from the Department of Clinical Epidemiology and Biostatistics for their invaluable contributions to the statistical analysis. Furthermore, the authors wish to convey heartfelt thanks to all the participants in this study.

### Conflict of Interest

The authors declare that they have no conflict of interest.
